# Supplementary material for: MetaRibo-Seq measures translation in microbiomes
Source: Nat Commun. 2020 Jun 29;11:3268. doi: 10.1038/s41467-020-17081-z (PMC7324362; doi:10.1038/s41467-020-17081-z)
Supplement: Supplementary file 10 — Supplementary Data 7 [file 41467_2020_17081_MOESM10_ESM.zip › File2/Confidence_High_Taxonomy/368081_out.krona.html]

Javascript must be enabled to view this page.

members
magnitude
magnitudeUnassigned
count
unassigned
taxon
rank

368081\_out

82

superkingdom
82
2

976
82
phylum

82
200643
class

order
82
171549

1
2005525
family

375288
1
genus

species
1
46503

SRS147139\_contig\_number\_17230

815
81
family

81
816
genus


SRS063985\_contig\_number\_30037
357276
1
species

species
78
817

SRS012902\_contig\_number\_79SRS013098\_contig\_number\_11516SRS013158\_contig\_number\_126SRS013215\_contig\_number\_34SRS014459\_contig\_number\_13858SRS014613\_contig\_number\_13592SRS014736\_contig\_number\_7082SRS015065\_contig\_number\_16667SRS015264\_contig\_number\_10948SRS015890\_contig\_number\_3415SRS015960\_contig\_number\_16759SRS016267\_contig\_number\_2266SRS016495\_contig\_number\_15116SRS016753\_contig\_number\_1683SRS017433\_contig\_number\_8596SRS017521\_contig\_number\_31634SRS017622\_contig\_number\_199SRS018313\_contig\_number\_7912SRS018656\_contig\_number\_9395SRS018984\_contig\_number\_20035SRS019267\_contig\_number\_2354SRS019397\_contig\_number\_2971SRS019601\_contig\_number\_12635SRS020869\_contig\_number\_22287SRS023346\_contig\_number\_6705SRS023829\_contig\_number\_90SRS023971\_contig\_number\_289SRS024075\_contig\_number\_2488SRS024132\_contig\_number\_10714SRS024388\_contig\_number\_1089SRS024549\_contig\_number\_463SRS043701\_contig\_number\_9637SRS046502\_contig\_number\_10644SRS046712\_contig\_number\_12SRS048981\_contig\_number\_727SRS049402\_contig\_number\_8748SRS051610\_contig\_number\_3628SRS053214\_contig\_number\_1196SRS053573\_contig\_number\_152SRS055966\_contig\_number\_236SRS056259\_contig\_number\_11855SRS057717\_contig\_number\_959SRS058723\_contig\_number\_6374SRS063127\_contig\_number\_1264SRS063190\_contig\_number\_10376SRS064276\_contig\_number\_25179SRS074964\_contig\_number\_3604SRS075021\_contig\_number\_591SRS075398\_contig\_number\_116SRS076804\_contig\_number\_14455SRS077194\_contig\_number\_5275SRS077335\_contig\_number\_1624SRS077589\_contig\_number\_1055SRS077730\_contig\_number\_1855SRS098644\_contig\_number\_2500SRS098717\_contig\_number\_752SRS1041112\_contig\_number\_135SRS104311\_contig\_number\_28602SRS104485\_contig\_number\_566SRS104636\_contig\_number\_2293SRS1054928\_contig\_number\_380SRS1055069\_contig\_number\_5533SRS140492\_contig\_number\_3910SRS140513\_contig\_number\_9348SRS143342\_contig\_number\_11246SRS143598\_contig\_number\_12675SRS144135\_contig\_number\_33814SRS144135\_contig\_number\_35888SRS144362\_contig\_number\_5041SRS146812\_contig\_number\_53442SRS147139\_contig\_number\_23658SRS147272\_contig\_number\_43SRS147377\_contig\_number\_5905SRS147557\_contig\_number\_30199SRS147653\_contig\_number\_6992SRS148091\_contig\_number\_460SRS148159\_contig\_number\_50343SRS893170\_contig\_number\_766

species

SRS021484\_contig\_number\_12722SRS1055034\_contig\_number\_989
2
818
